# Supplementary figures and images for: Trichiasis with and without tarsal conjunctival scarring: A multi-country observational study
Source: PLOS Glob Public Health. 2025 Oct 21;5(10):e0004356. doi: 10.1371/journal.pgph.0004356 (PMC12539719; doi:10.1371/journal.pgph.0004356)

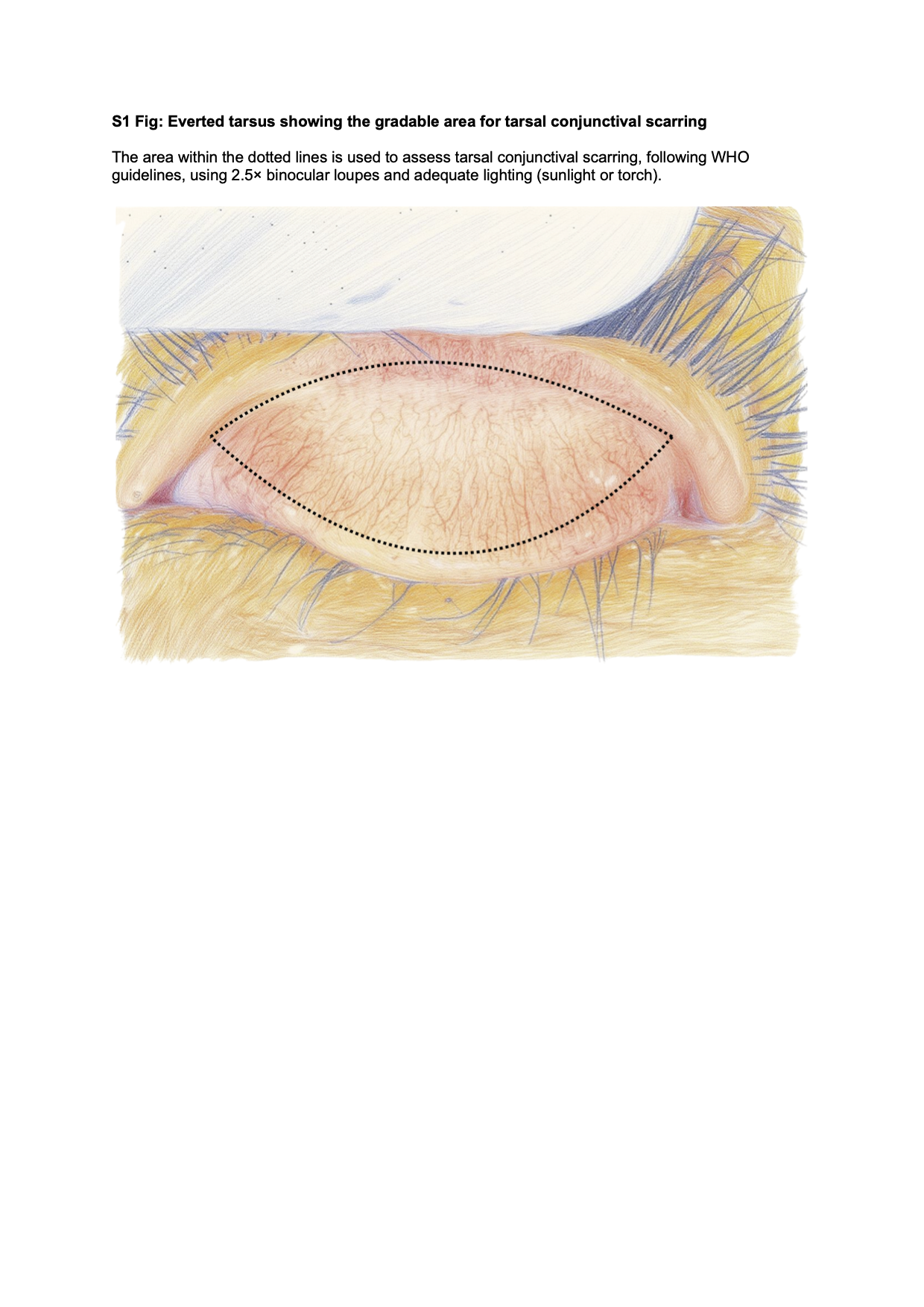

Supplement: S1 Fig — (TIF) [file pgph.0004356.s001.tif]
