# Supplementary material for: Trichiasis with and without tarsal conjunctival scarring: A multi-country observational study
Source: PLOS Glob Public Health. 2025 Oct 21;5(10):e0004356. doi: 10.1371/journal.pgph.0004356 (PMC12539719; doi:10.1371/journal.pgph.0004356)
Supplement: Attachment — Submitted filename: PLOS Reviwer comments.docx [file pgph.0004356.s003.docx]

**The following point needs to be review**

1. Abstract should be little bit short

2.Review the introduction and background section to understand the context and significance of the study

3.assess the sampling strategy and data methods to determine if they are appropriate for the research question

4 evaluate the data analysis methods , result and conclusion drawn from the data . check for any errors or inconsistencies

5 the section related to trichiasis and tarsal conjunctival scarring to ensure that the definition , classification and assessment methods are clear and consistent

6. Assess the strength and limitation of the multi country observational study design. evaluate the generalizability of the findings across different countries and population
