# Supplementary material for: Trichiasis with and without tarsal conjunctival scarring: A multi-country observational study
Source: PLOS Glob Public Health. 2025 Oct 21;5(10):e0004356. doi: 10.1371/journal.pgph.0004356 (PMC12539719; doi:10.1371/journal.pgph.0004356)
Supplement: Attachment — Submitted filename: Response to Editors Requests_21May25 .docx [file pgph.0004356.s004.docx]

1. **Thank you for providing the requested questionnaire for global inclusivity. However, we note that the questionnaire has been submitted as an 'Other' file type. Kindly resubmit the global inclusivity questionnaire as 'Supporting Information' file.**

The global inclusivity questionnaire has been submitted as 'Supporting Information' file.

2. We note your Competing Intersect statement:

“I have read the journal's policy and the authors of this manuscript have the following competing interests: Michaela Kelly, Caleb Mpyet and Tom Millar are the employee of Sightsavers International, UK. Emma Harding-Esch and Ana Bakhtiari receive salary support from the International Trachoma Initiative, which receives an operating budget and research funding from Pfizer Inc., the manufacturers of Zithromax® (azithromycin). Emma Harding-Esch, Ana Bakhtiari and Caleb Mpyet are members of the core Tropical Data team, and Caleb Mpyet and Gilbert Baayenda are Tropical Data principal trainers. No conflicting relationship exists for any other author.”

Before we proceed, please clarify whether the author's commercial affiliations alter your adherence to PLOS ONE policies on sharing data and materials. If not, please state the following: “This does not alter our adherence to PLOS ONE policies on sharing data and materials.”

Once we receive this information, we will update your Competing Interests statement on your behalf.

Thanks. I confirm that the author's commercial affiliations do not alter our adherence to PLOS ONE policies on sharing data and materials.

3. Can you please upload a copy of your latest revised manuscript (clean copy) that does not contain any tracked changes, comments, or highlighting.

I have attached it as a PDF as the figure between lines 274- 275 is creating automatic field code change resulting the tracked changes in the docx file. I hope this ok.

4. Some material included in your submission may be copyrighted. According to PLOS’s copyright policy, authors who use figures or other material (e.g., graphics, clipart, maps) from another author or copyright holder must demonstrate or obtain permission to publish this material under the Creative Commons Attribution 4.0 International (CC BY 4.0) License used by PLOS journals. Please closely review the details of PLOS’s copyright requirements here: PLOS Licenses and Copyright. If you need to request permissions from a copyright holder, you may use PLOS's Copyright Content Permission form.

Please respond directly to this email or email the journal office and provide any known details concerning your material's license terms and permissions required for reuse, even if you have not yet obtained copyright permissions or are unsure of your material's copyright compatibility.

Potential Copyright Issues:

Figure 2: Please confirm (a) that you are the photographer; or (b) provide written permission from the photographer to publish the photo(s) under our CC-BY 4.0 license.

I am not the photographer. But I am the Chief Investigator that trained and hired the photographers to take the tarsal conjunctival photos in this project using our project devices. The photographer’s role was just to take the phots and they were paid salary from the project for that work. We, the leads of this project, own the photos. Therefore, we the authors hold the copy right for all the photos included in this manuscript and provide to your journal permission to publish them under the Creative Commons Attribution 4.0 International (CC BY 4.0) License.
