# Supplementary material for: Trichiasis with and without tarsal conjunctival scarring: A multi-country observational study
Source: PLOS Glob Public Health. 2025 Oct 21;5(10):e0004356. doi: 10.1371/journal.pgph.0004356 (PMC12539719; doi:10.1371/journal.pgph.0004356)
Supplement: Attachment — Submitted filename: reviwer PLOS Comments.docx [file pgph.0004356.s005.docx]

Comments

1. Add more keywords
2. Clarify inclusion/exclusion criteria
3. Mention Standardize scarring grading
4. Address potential biases
5. Provide detailed methodology
6. Write the Implications for trachoma control
